# Optimal dose and duration of iron supplementation for treating iron deficiency anaemia in children and adolescents: A systematic review and meta-analysis

**DOI:** 10.1371/journal.pone.0319068

**Published:** 2025-02-14

**Authors:** Tanveer Rehman, Ritik Agrawal, Farhad Ahamed, Saibal Das, Srijeeta Mitra, Dinesh Kumar, Chinmayee Sethy, Srikanta Kanungo, Debdutta Bhattacharya, Sanghamitra Pati

**Affiliations:** 1 ICMR Regional Medical Research Centre, Bhubaneswar, Odisha, India; 2 Model Rural Health Research Unit, Namkum, Ranchi, Jharkhand, India; 3 All India Institute of Medical Sciences, Kalyani, West Bengal, India; 4 ICMR-Centre for Ageing and Mental Health, Kolkata, India; 5 Department of Global Public Health, Karolinska Institute, Stockholm, Sweden; 6 Indian Institute of Public Health, Gandhinagar, Gujarat, India; North-Caucasus Federal University, RUSSIAN FEDERATION

## Abstract

**Introduction:**

Iron deficiency anaemia (IDA) accounts for nearly two-thirds of all anaemia cases globally. Despite the widespread use of iron supplementation, the optimal dose and duration for treating IDA remain unclear. In this study, we aimed to determine the most effective dose and duration of iron supplementation for improving haemoglobin (Hb) levels in children and adolescents (≤19 years) with IDA.

**Methods:**

A systematic review and meta-analysis were conducted. We searched MEDLINE, Embase, CINAHL, and the Cochrane Library for peer-reviewed studies published between 2013 and 2024. The interventions included iron supplementation with a defined dose and duration of at least 30 days. Comparators were placebo, no treatment, or alternative regimens. The outcome was the change in Hb levels. Eligible studies included IDA cases diagnosed through ferritin level measurements in healthy individuals. Studies involving pregnant women or children with underlying conditions were excluded. A meta-analysis was performed using standardized mean differences to pool effect sizes for Hb improvement with 95% confidence intervals (CIs). Subgroup analyses were performed for different treatment durations (<3 months, 3–6 months, >6 months) and dosage categories (<5 mg/kg/day, 5–10 mg/kg/day, >10 mg/kg/day). A random-effects meta-regression model was used to determine the optimal dose and duration, accounting for known covariates affecting Hb improvement.

**Results:**

A total of 28 studies with 8,829 participants from 16 countries were included. The pooled effect size for Hb improvement was 2.01 gm/dL (95% CI: 1.48–2.54, *p* < 0.001). Iron supplementation for less than 3 months showed the highest significant effect size (2.39 gm/dL, 95% CI: 0.72–4.07), followed by treatments exceeding 6 months (1.93 gm/dL, 95% CI: 0.09–3.77). The lowest effect size was observed in treatments lasting 3–6 months (1.58 gm/dL, 95% CI: 0.93–2.23). Low-dose iron supplementation (<5 mg/kg/day) demonstrated favourable trends in Hb improvement, particularly in individuals with lower baseline Hb levels. Oral ferrous sulphate had a significant effect (2.03 gm/dL, 95% CI: 1.24–2.82), while parenteral ferric Carboxymaltose showed consistent efficacy.

**Conclusion:**

Low-dose iron supplementation (<5 mg/kg/day) combined with treatment durations of either less than 3 months or more than 6 months, is optimal for improving Hb levels in children and adolescents with IDA. Tailoring treatment based on baseline Hb levels and anaemia severity is essential. These findings provide evidence to support updated guidelines on iron supplementation in paediatric and adolescent populations and inform national anaemia management programmes.

**Trial registration:**

**Prospero registration number**: This study was registered with PROSPERO (CRD42024541773).

## Introduction

Anaemia affects approximately 1.92 billion people, accounting for 24.3% of the world’s population [1]. Its prevalence is particularly high in low-and middle-income countries (LMICs) due to nutritional deficiencies, infectious diseases, and limited access to healthcare. The economic burden of anaemia is substantial, including decreased productivity, increased healthcare costs, and reduced educational outcomes [2, 3]. Among the various types of anaemia, iron deficiency anaemia (IDA) is the most prevalent, contributing to 66.2% of all anaemia cases and is the leading cause of anaemia-related years lived with disability [1]. During childhood and adolescence, iron requirements increase due to rapid growth and development, making these age groups particularly vulnerable to IDA. Children under the age of five have a high prevalence of anaemia, at 41.4% [4]. Iron deficiency in children is associated with adverse psychomotor, cognitive, and socioemotional development, which can have long-lasting effects on adolescent behaviour and development, even after the deficiency is corrected [5–8]. Adolescents are similarly vulnerable, with a global anaemia prevalence of 15% and significant disparities between developed (6%) and developing countries (27%) [9, 10].

While most individuals with mild to moderate IDA (96.6%) may not experience severe complications, approximately 3.4% of untreated or severe cases may face significant health risks [11, 12]. Common complications include cardiovascular issues such as heart failure and left ventricular hypertrophy, which can affect up to 20–30% of individuals with severe IDA [13, 14]. Early diagnosis and timely intervention are crucial in preventing such outcomes. Recommended strategies include iron supplementation, dietary diversification, food fortification, and home-based interventions [15].

IDA is commonly diagnosed by assessing serum ferritin levels (<15 μg/L) and age- and sex-specific haemoglobin (Hb) thresholds, as outlined by the World Health Organization (WHO) [16, 17]. Iron supplementation is a critical intervention for both the prophylactic management and treatment of IDA, aiming to replenish iron stores essential to produce healthy red blood cells [18]. Oral iron supplementation is effective when intestinal absorption is intact, making it a viable treatment option for mild to moderate anaemia. Intravenous administration, though preferred for rapid repletion, is generally reserved for severe cases.

Iron supplementation typically involves either inorganic forms, such as ferrous sulfate, or organic forms, such as iron ascorbate or lysinate. While organic forms offer superior absorption and reduced toxicity, they are often costlier and less accessible, especially in resource-limited settings [19]. In contrast, inorganic forms such as ferrous fumarate (33% elemental iron), ferrous sulfate (20%), and ferrous gluconate (12%) are widely used due to their affordability and availability [20, 21]. Despite concerns about their lower bioavailability and higher potential for gastrointestinal side effects, inorganic iron formulations, particularly ferrous sulfate, are recommended in most clinical guidelines and public health programs.

For prophylactic management, WHO recommends three consecutive months of iron supplementation, administered either daily or intermittently depending on the local prevalence of anaemia [22–26]. For children aged 6–23 months, the recommended dose is 10–12.5 mg of elemental iron, increasing to 30 mg for children aged 24–59 months, and to 30–60 mg for adolescents aged 5–19 years. In therapeutic contexts, dosing regimens may vary: a typical treatment involves 65 mg of elemental iron taken thrice daily, though lower dosages (e.g., 40 mg twice daily) are equally effective and associated with fewer side effects [18]. Complete normalization of Hb levels may take up to three months, while full replenishment of iron stores potentially requires even longer [20, 27]. In India, the Intensified National Iron Plus Initiative (I-NIPI) recommends 3 mg of iron per kg of body weight daily for children aged 6 months to 9 years, and two tablets (each containing 60 mg elemental iron) daily for adolescents aged 10–19 years [28]. In other countries, the recommended dose ranges from 3–6 mg of iron per kg daily for all ages, with supplementation continued for three months after Hb levels normalize [29, 30].

Despite the widespread use of iron supplementation, there is no global consensus on the optimal dose, schedule, and duration necessary to achieve and sustain recovery from anaemia in children and adolescents [22, 23]. Current WHO guidelines for iron supplements are limited by a lack of high-quality evidence, resulting in significant variability in clinical practices [31]. The quality of evidence for the dose and duration of IDA treatment, as assessed using the Grading of Recommendations Assessment, Development and Evaluation (GRADE) methodology, varies from high to low [22, 23, 32]. Given that most individuals with IDA do not experience complications [11], generating evidence regarding the appropriate dosage and duration for uncomplicated cases is critical. To address this knowledge gap, we aimed to determine the optimal dosing and duration of iron supplementation for the treatment of IDA in children and adolescents.

## Methods

### Design and registration

We conducted a systematic review and meta-analysis of original observational and interventional studies. This review was registered with PROSPERO (CRD42024541773) [33] and conducted in adherence to PRISMA guidelines (S1 Table) [34].

### Information sources and search strategy

The literature search was conducted initially on May 15, 2024, with updated searches on July 21, 2024. The peer-reviewed databases that were searched included MEDLINE (via PubMed), Embase (via Ovid), CINAHL (via EBSCO) and Cochrane Library. The basic search syntax utilised in the study comprised three main concepts: child or adolescent, anaemia (with IDA included), and iron supplementation regimens. The search was restricted to English-language studies involving participants aged ≤19 years. Broader terms such as “anaemia” and “iron regimens” were included to ensure a comprehensive capture of relevant literature. Reference lists of included studies were manually checked for additional relevant papers. Systematic review experts (SD, FA) assisted in refining the search strategy. A detailed explanation of the methodology is provided in S2 Table.

### Eligibility criteria

The study inclusion criteria were decided a priori and are presented according to the PICO framework.

- Populations: children and adolescents (≤ 19 years) diagnosed with IDA
- Interventions: Iron supplementation with a defined dose and duration of at least 30 days.
- Comparators: Placebo or no treatment, different doses or durations of iron supplementation, standard treatment or alternative interventions
- Outcome: Change in Hb levels

Eligible studies comprised publications in or after 2013 and reported the length of duration of the treatment of IDA among children and adolescents. Randomised controlled trials (RCTs), quasi-experimental studies, prospective and retrospective cohort studies, case-control studies, analytical cross-sectional studies, and observational studies were eligible for inclusion. Commentaries, editorials, review papers, case studies, studies with no data presented, and conference abstracts were excluded. We included IDA cases that were diagnosed through measurements of ferritin levels in healthy individuals. Studies involving pregnant women and children with underlying medical conditions that could influence anaemia progression (e.g., genetic disorders, chronic illness) were not considered for the present study [35–37]. These conditions were excluded to ensure the results accurately reflected IDA treatment in otherwise healthy individuals.

### Study selection

The results of the peer-reviewed searches from all the databases were first imported to the Mendeley reference manager for deduplication and then exported to Rayyan for primary screening [38, 39]. The study selection process involved three stages:

1. Primary screening: Two independent investigators performed preliminary screening of the title, abstract, and keywords (inter-rater agreement = 88–95%) (RA, SM, CS, and TR). Disagreement between reviewers was resolved via discussion, and in cases in which consensus was not reached, a third reviewer was consulted. Full-text articles were retrieved for the studies shortlisted based on the eligibility criteria.
2. Secondary screening: The same investigators screened the full text of these retrieved studies and assessed them against the review’s eligibility criteria (inter-rater agreement = 95–96%).
3. Final selection: After secondary screening, a final consensus on study inclusion was reached with input from all investigators (FA, SD).

### Data collection and data items

Studies were extracted into a pre-designed data extraction Excel sheet (by RA and SM). Extractions were double-checked by a second, different team member (TR, FA, or SD), and conflicts were resolved between the extractor and double-checked by discussion. For studies with incomplete or missing data on key outcomes, efforts were made to contact the corresponding author (or authors) of the study to request additional data. Authors were contacted up to three times over a six-week period, and responses were recorded. If no response was received, the study was included in the analysis based on the available data, provided that missing information did not critically affect the study’s eligibility or primary outcome measures. We stopped contacting authors for additional information or data on August 11, 2024.

For the present study following data were extracted: for general characteristics of the study, author information, study title and year of publication were extracted. For the methods section, the information on study design, study period, study setting (community/workplace/facility), community (urban/rural), geographical region, country, sample size, sampling technique, Hb diagnostic criteria, dose and duration of different iron regimens given for the treatment of IDA was obtained. For estimating the outcome, Hb change across arms from baseline was extracted from the literature.

### Synthesis methods

Meta-analysis was performed using R (V.3.0.3 (2014-03-04), The R Foundation for Statistical Computing, Vienna, Austria) [40]. The effect size for improvement in Hb between the intervention and control group was calculated by using the standardised mean differences (SMD) and standard errors (SE) for each study to obtain the pooled effect estimate with 95% confidence intervals (CIs). Doses were standardized to mg/kg/day based on the WHO growth chart and the National Centre for Health Statistics (NCHS) stature-for-age chart [41–44]. Forest plots were used to present pooled effect sizes. We used the random-effects model with the restricted maximum likelihood method to account for the clinical and methodological heterogeneity across the included studies. The heterogeneity was assessed using the χ^2^ test on Cochrane’s Q statistic, with the I^2^ statistic used to quantify the degree of heterogeneity. I^2^ values of 25%, 50%, and 75% represented low, moderate, and high heterogeneity, respectively [45].

Since Hb change was the primary outcome, estimating the optimal duration and dose was challenging. Given the anticipated heterogeneity, subgroup analyses were performed by route of administration (oral vs parenteral), dosage (<5 mg/kg/day, 5–10 mg/kg/day, >10 mg/kg/day), and duration (<3 months, 3–6 months, >6 months) [46]. To explore potential factors influencing Hb improvement, meta-regression was performed on covariates with a p-value <0.2 in univariate models [47, 48].

To determine the optimal dose and duration of iron supplementation, we adjusted for several covariates that could influence Hb improvement:

**(1) Age**: Iron requirements vary across different age groups.
**(2) Study setting**: Differences in lifestyle, diet, and access to healthcare in urban versus rural settings.
**(3) Iron regimens**: Both oral and intravenous routes were considered.
**(4) Mean baseline Hb**: Higher initial Hb levels may lead to smaller improvements
**(5) Gender**: Gender-specific differences in iron metabolism were accounted for.
**(6) Dose (mg/kg/day)**: Dosage was adjusted to evaluate its impact on Hb improvement.
**(7) Duration (months)**: To identify the optimal length of treatment.

Missing data were not imputed, and sensitivity analyses were conducted to assess the potential impact of excluding incomplete data. Sensitivity analysis has also been done to assess the robustness of the results by removing the studies one at a time and evaluating their influence on the pooled estimates. Publication bias was assessed using funnel plots and asymmetry was tested using Egger’s test (*p* < 0.10) [49, 50].

### Risk of bias and GRADE assessment

The risk of bias was independently assessed by three reviewers (RA, CS, SD) using the Joanna Briggs Institute (JBI) critical appraisal checklists for RCTs [51] and cohort studies [52]. Disagreements were resolved through discussion, and studies were rated as good, fair, or poor based on their methodological quality. A summary of study quality assessments is provided in S3 Table.

We used the GRADE approach to evaluate the overall quality of evidence for each outcome [53]. It assesses five key domains: risk of bias, inconsistency, indirectness, imprecision, and publication bias [54]. Outcomes were rated on a scale from 1+ (low quality) to 4+ (high quality) [55]. Two independent reviewers (RA and SM) conducted the GRADE assessment, resolving any disagreements through consensus or by consulting a third reviewer (SD). We employed GRADEpro GDT software to summarise the findings and generate tables with relative and absolute effects and the corresponding certainty of evidence [56, 57]. The overall certainty was classified as high, moderate, low, or very low, indicating our level of confidence in the effect estimates [58]. While the JBI tool primarily focuses on the internal validity and methodological quality of individual studies, GRADE considers a wider perspective by assessing the overall quality of evidence across all relevant studies for each outcome [59].

## Results

### Literature search and selection

**Fig 1:**
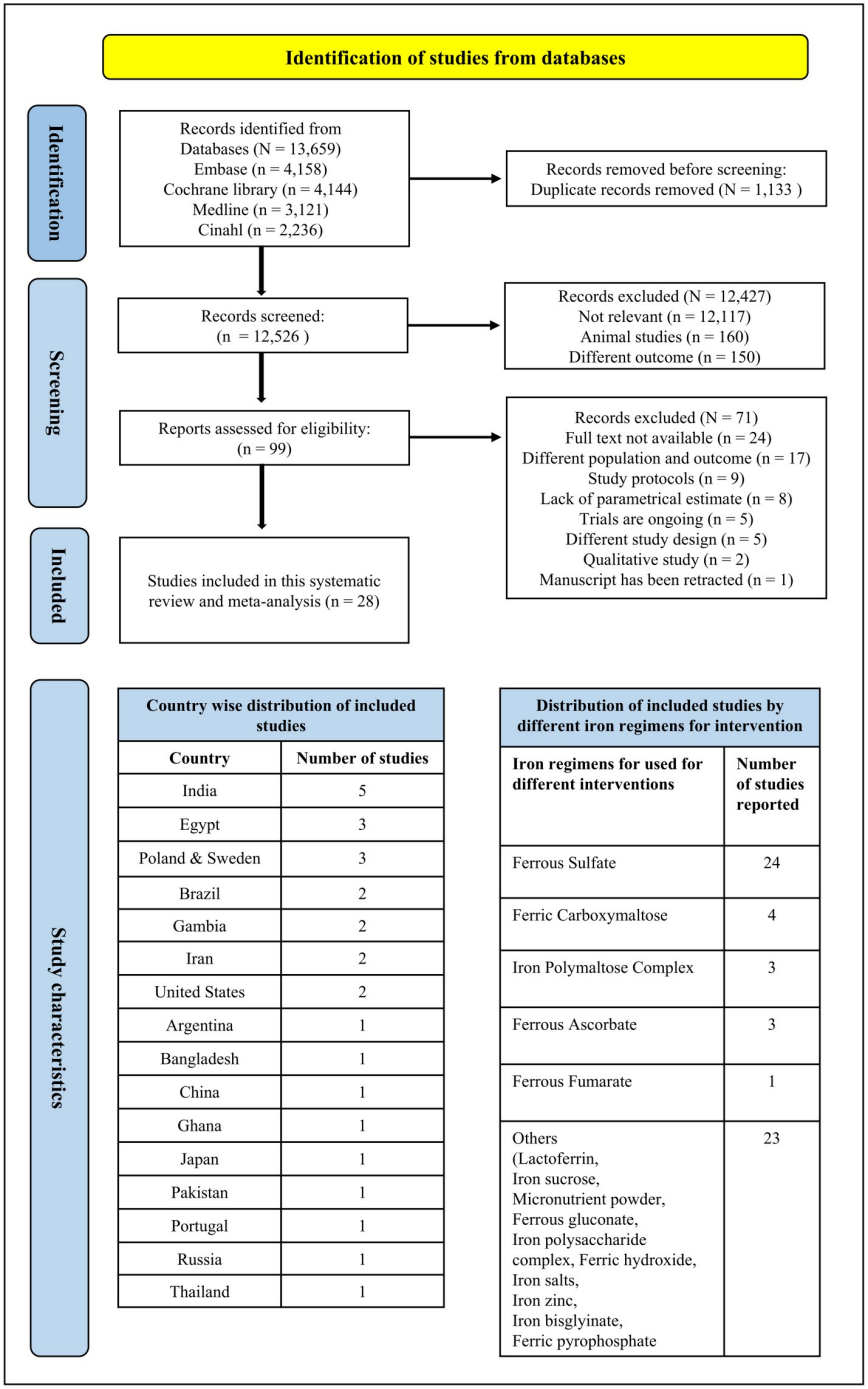
PRISMA flow diagram illustrating the study selection process for this systematic review.

A total of 13,659 papers published since 2013 were identified in our searches (Fig 1). Of these, 99 articles met our inclusion criteria, and 28 studies were finally included in the analysis. The details of all the studies identified with their reason for exclusion are provided in the S4 Table. These studies spanned 16 countries and included 8,829 children & adolescents aged 1 month to 19 years [60–87] (Table 1). The PRISMA flow diagram depicting the study selection procedure is given in Fig 1.

**Table 1:** Characteristics of all included studies in the systematic review.

SN	Author name	Year of study conducted	Country	Study design	Study setting	Age group	Mean age (SD age)	Sample size	Anaemia diagnosed method
1	Pasricha et al. [60]	2017	Bangladesh	Double-blind, a three-group, individually randomized placebo-controlled trial	Rural	7.5 to 8.5 months	8 (0.3) months	3300	Defined as Hb level of <11 gm/dL, and iron deficiency as ferritin level of <12 mcg/L or <30 mcg/L if the CRP level was >5 mg per litre
2	Falahati et al. [61]	2020	Iran	Randomized single-blind clinical trial	Urban	6 to 24 months	4.6 (2.4) months	120	Anaemia defined as Hb<11.0 gm/dL Iron Deficiency defined as ferritin level<10 mg/L or MCV<70 fL and RDW>14.5%
3	Fujita et al. [62]	2017	Japan	Randomised clinical trial	NA	9 to 48 months	NA	80	Infants and Young children (aged 6–24 months) have IDA, Hb levels < 11.0 gm/dL and Serum Ferritin levels < 12 mcg/L
4	Bah & Stelle et al. [63]	2021	Gambia	A placebo-controlled, randomized, double-blind trial	Rural	6 to 10 weeks	NA	101	NA
5	Moradveisi et al. [64]	2019	Iran	Interventional single-blind study	NA	6 months to 4 years	NA	88	Hb level of more than 5 and less than 11 gm/dL, ferritin <12 mcg/L or transferrin saturation <15%
6	Svensson et al. [65]	2015	Poland & Sweden	Randomized, double-blind, placebo-controlled trial	NA	Infants born at term and Intervention at 4 months of age	NA	221	Hb levels <10.5 gm/dL
7	Varea et al. [66]	2017	Argentina	Randomized, controlled clinical trial	Urban	3 to 6 months	NA	275	Hb < 9.5 gm/dL at 3 months of age and < 11.0 gm/dL at 6 months of age, ID: ferritin < 12 ng/mL, and IDA: Hb < 11 gm/dL and ferritin < 12 ng/mL.
8	Chirdkiatgumchai et al. [67]	2016	Thailand	Double-blind randomized controlled trial	NA	6 to 18 years	NA	52	Serum ferritin level <30 mg/dL or transferrin saturation [serum iron/total iron binding capacity *100] <16%.
9	Amrousy et al. [68]	2020	Egypt	Randomized controlled trial	NA	5 to 18 years	11.2 (2.4) years	80	Hb<11.5 gm/dL for children aged 5–11 years, < 12 gm/dL for children 12–14 years and females of 15 years or more, < 13 gm/dL for males of 15 years or more
10	Hamed et al. [69]	2019	Egypt	Randomized controlled trial	Urban	5 to 13 years	10.9 (3.3) years	160	Hb <11 gm/dL; Serum ferritin ≤30 mg/l. Transferrin saturation ≤16%; MCV ≤75 fL; MCHC ≤32 gm/L)
11	Sourabh et al. [70]	2018	India	Non-randomized intervention study	Urban	<5 years	3.8 (2.0) years	7	Hb <11 gm/dL for children between 6 months-59 months; Hb <11.5 gm/dL for children aged 60 months-11 years
12	Gupta et al. [71]	2017	India	Cluster randomized control trial	Rural	12 to 19 years	14.5 (1.8) years	760	Moderate anaemia (8.0–10.9 g/dL) and Mild anaemia (11.0–11.9 g/dL)
13	Gupta et al. [72]	2013	India	Randomized controlled trial	Both	10 to 19 years	14.3 years	331	NA
14	Azevedo et al. [73]	2014	Portugal	Randomized controlled trial	Urban	5 to 19 years	NA	19	Anaemia was classified according to WHO Criteria: 11.5 gm/dL in children aged between 5 to 11 years, 12.0 gm/dL for girls >12 years old, and 13 gm/dL for boys >12 years old.
15	Bakht et al. [74]	2022	Pakistan	Randomized controlled trial	Both	6 to 12 years	8.9 (1.9) years	148	Children from both genders between 6–12 years of age with Hb below 10 gm/dL and ferritin level below 7 micro gm/dl were included
16	Mohammed et al. [75]	2017	Gambia	Three-arm parallel individually randomized placebo-controlled double-blind study	Rural	6 to 35 months	NA	642	Hb <11 gm/dL and ferritin <30 mcg/l
17	Yun et al. [76]	2020	China	Randomized controlled trial	Urban	6 to 34 months	NA	147	Hb ≤ 9 gm/dL to <11 gm/dL
18	Kamal et al. [77]	2021	Egypt	Prospective intervention study	Urban	>2 years	NA	150	Iron deficiency anaemia: decreased serum ferritin, decreased serum iron and decreased Hb.
19	Fisyun et al. [78]	2019	Russia	Randomized controlled trial	Both	1 month to 18 years	2.2 (1.1) years	65	WHO criteria for diagnosing anaemia (2008): decrease in Hb level < 11 gm/dL at the age of 6 months—4 years 11 months; <11.5 gm/dL at the age of 5 years—11 years 11 months and <12 gm/dL at the age of over 12 years.
20	Powers and Mark et al. [79]	2015	United States	Randomized controlled trial	Urban	9 months to 20 years	14.6 (8.9) years	87	WHO criteria for diagnosing iron deficiency anaemia.
21	Gosdin et al. [80]	2017	Ghana	Pre-post Longitudinal Program Evaluation	Both	10 to 19 years	14.8 (6.7)	1521	Capillary blood was used to measure Hb using a Hemocue 301 10–11 years: Hb<11.5 gm/dL ≥12 years: Hb<12 gm/dL
22	Matos et al. [81]	2016	Brazil	Cluster randomized clinical trial	Urban	6 to 18 months	11.3 (2.5) months	97	As per the WHO criteria, Hb concentration <11.0 gm/dL was used as a cutoff point to define anaemia.
23	Kaushik et al. [82]	2020	India	Open labelled randomized study	Both	6 to 24 months	14.3 (0.7)	103	Anaemia defined as Hb less than 11 gm/dL
24	Powers and George et al. [83]	2015	United States	Double-blind superiority randomized clinical trial	Urban	9 to 48 months	23 (21) months	80	Hb < 10 gm/dL, MCV < 70 Fl, Reticulocyte Hb < 25 pg, Serum ferritin < 15 ng/ml, total iron binding capacity > 425 mcg/dl
25	Korczowski et al. [84]	2017	Russia and Poland	Phase 2, open-label, non-randomized, multicentre study	Both	1 to 17 years in Poland and 6 to 17 years in Russia	9.1 (6.1) years	35	Hb concentration <11 gm/dL and a transferrin saturation (TSAT) <20%.
26	Patil et al. [85]	2016	India	Single-centre open labelled randomized controlled trial	Both	1 to 12 years	2.6 (1.8) years	100	IDA diagnosis was based on the combination of at least two of the following criteria in addition to Hb <10 (g%) at baseline: lower than normal (80–100 fL), mean corpuscular volume (MCV), raised red cell volume distribution (RDW) than the normal range (11–15%) or low serum ferritin levels (<12 ng/ml but not >100 ng/ml)
27	Wegier et al. [86]	2019	Poland	Phase 3 single-arm Open-label trial	Both	6 to 53 months	10.4 (3.9) months	40	IDA: Hb (7–10.9 gm/dL) & serum ferritin <12 ng/ml
28	Name et al. [87]	2016	Brazil	Pilot randomized trial	Both	1 to 13 years	3.9 (1.2) years	20	Hb levels below the criteria for IDA stipulated by the WHO (children aged 6–59 months, <11.0 gm/dL; 5–11 years, <11.5 gm/dL; 12 years or older, <12.0 gm/dL)

### General characteristics of selected studies

The sample sizes of the included studies ranged from seven participants [65] to 3300 individuals [55]. Most were RCTs, with only three cohort studies [70, 73, 80]. The studies were geographically diverse, with five conducted in India [70–72, 82, 85], three in Egypt [68, 69, 77], and others in countries such as Brazil, the United States, Gambia, Iran, Poland, and Sweden [60–87]. As per the World Bank classification [88], one study was from a low-income country, five from LMICs, six from upper-middle-income countries, and four from high-income countries (HICs). A detailed description of all the studies included from different countries is provided in Fig 1. Of the 28 studies, 24 reported the use of ferrous sulfate, while four utilized iron poly-maltose complex or ferric carboxymaltose. The details of all the data extracted from included studies are provided in S5 Table.

### Variations of iron supplementation by geographical region

Of the 28 studies included in this review, most came from LMIC settings in Asia, Africa, and Latin America, such as India, Egypt, and Gambia, while some (n = 4, 14.3%) were conducted in HICs such as the United States, Poland, Japan and Sweden. Studies in LMICs commonly employed higher doses of iron (5–10 mg/kg/day), and supplementation periods often exceeded six months. In contrast, studies in HICs typically used lower doses (<5 mg/kg/day), prioritizing reduced adverse effects like gastrointestinal discomfort. Treatment durations were shorter (<3 months) and adhered to standard clinical protocols.

### The effect size for improvement in Hb

**Fig 2:**
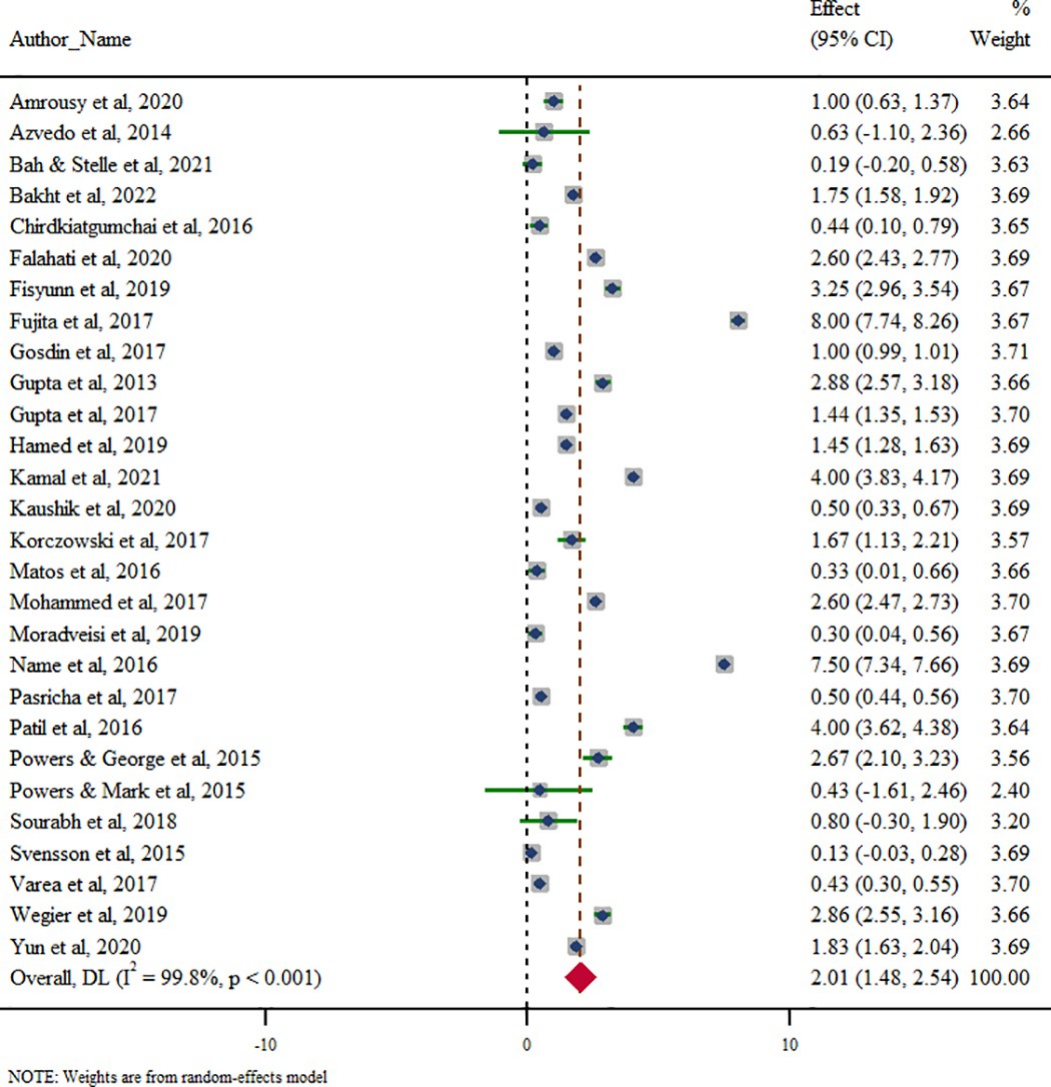
Meta-analysis of improvement in haemoglobin between intervention and control group (N = 28).

The combined effect size for improvement in Hb between the intervention and control group was 2.01 gm/dL (95% CI: 1.48–2.54, *p* < 0.001). However, significant heterogeneity was observed among studies (Cochrane Q = 12,933.8, *p* < 0.001; I^2^ = 99.80). The forest plot of combined effect size is given in Fig 2.

### Subgroup analysis based on duration of iron supplementation

The subgroup analysis based on the duration of iron supplementation showed the following differences in effect sizes:

- <3 months (N = 13): Highest effect size of 2.39 gm/dL (95% CI: 0.72–4.07, *p* < 0.001)
- 3–6 months (N = 13): Lowest effect size of 1.58 gm/dL (95% CI: 0.93–2.23, *p* < 0.001)
- >6 months (N = 2): Effect size of 1.93 gm/dL (95% CI: 0.09–3.77, *p* = 0.039)

**Fig 3:**
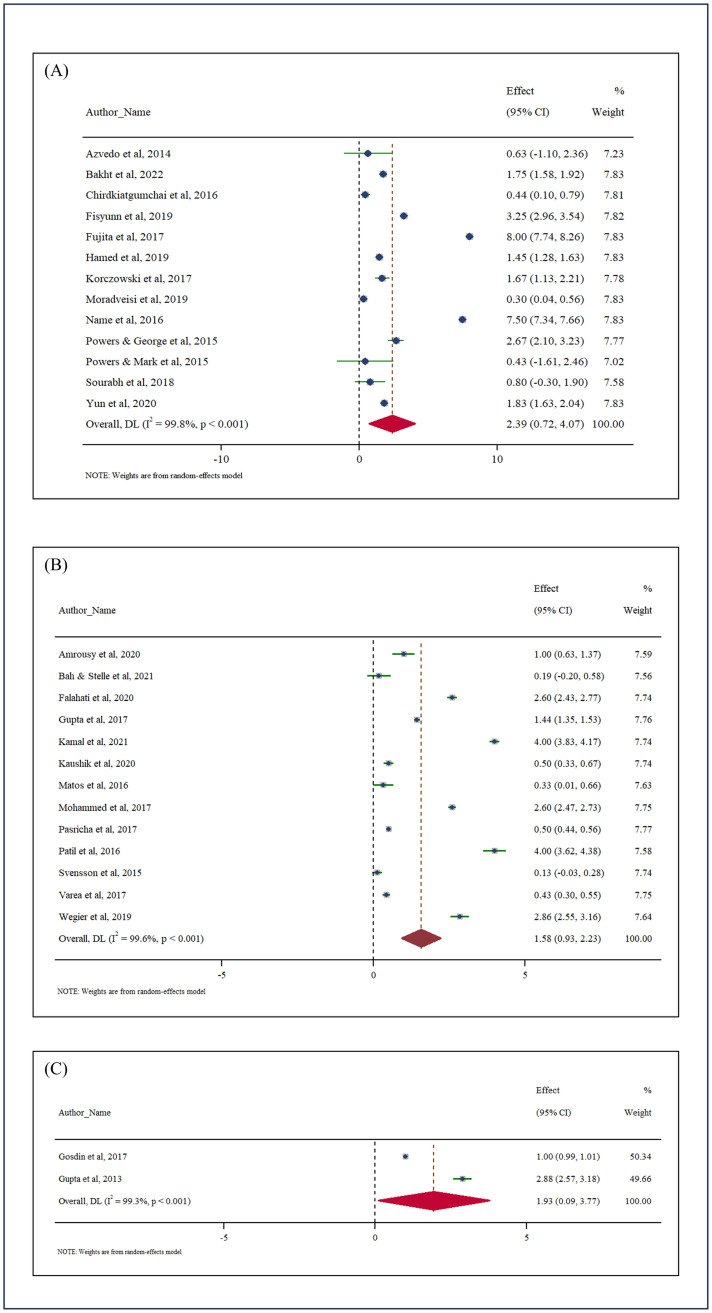
Subgroup meta-analysis for improvement in Hb between different intervals of iron administration: (A) For shorter duration: <3 months (N = 13), (B) For moderate duration: 3–6 months (N = 13), (C) For longer duration: >6 months (N = 2).

Heterogeneity remained substantial in all subgroups, with I^2^ values of 99.8% for <3 months, 99.6% for 3–6 months, and 99.3% for >6 months. The detailed description is given in Fig 3.

### Optimal dose and duration for iron supplementation

After adjusting for all the known covariates which can affect the improvement in Hb, iron supplementation for less than three months was associated with a significant positive effect on Hb levels (coefficient: 5.32, 95% CI: 0.23 to 10.41, *p* = 0.04). Similarly, supplementation for more than six months showed a significant positive effect (coefficient: 5.28, 95% CI: 0.47 to 10.41, *p* = 0.03). In contrast, a duration of 3–6 months was associated with a significant reduction (coefficient: -2.66, 95% CI: -5.20 to -0.11, *p* = 0.04). Additionally, the low dose regimen (<5 mg/kg/day) was associated with an increase in effect size of 1.76 units (95% CI: -0.53 to 4.05, *p* = 0.12).

### Subgroup analysis based on route of administration and regimens

The overall effect size (N = 26) for both parenteral and oral routes was 1.79 gm/dL (95% CI: 1.08–2.50). Parenteral ferric carboxymaltose had a relatively low level of heterogeneity (I^2^ = 24.7%, *p* < 0.001) and a significant effect of 1.45 gm/dL (95% CI: 0.54–2.37). Oral regimens displayed greater variability:

- Ferrous Ascorbate: Highest effect size of 2.44 gm/dL (95% CI: -0.69–5.58, I^2^ = 96.6%, *p* < 0.001)
- Ferrous Sulfate: Effect size of 2.03 gm/dL (95% CI: 1.24–2.82, I^2^ = 99.7%, *p* < 0.001).
- Other Iron Regimens: Effect size of 2.28 gm/dL (95% CI: 0.50–4.07, I^2^ = 99.9%, *p* < 0.001).

**Fig 4:**
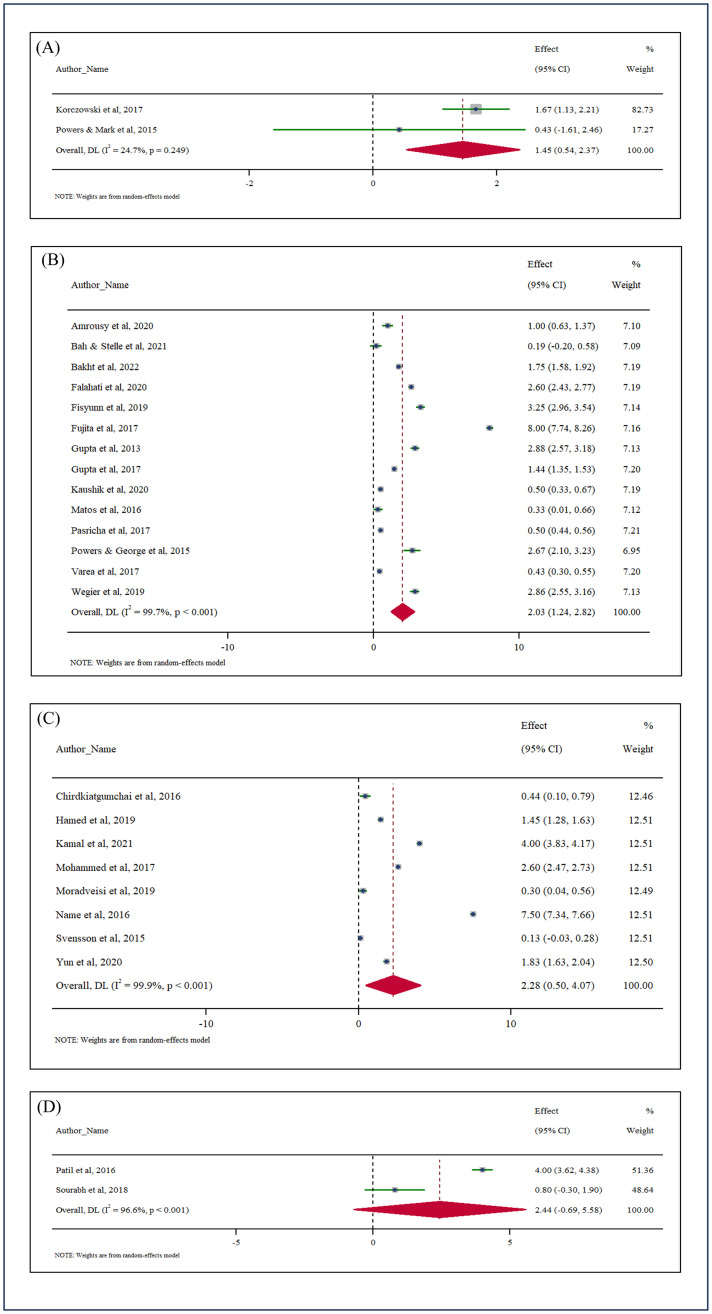
Subgroup meta-analysis for improvement in Hb between different iron regimens: (A) Parenteral Ferric Carboxymaltose (N = 2), (B) Oral Ferrous Sulfate (N = 14), (C) Oral Others (N = 8), (D) Oral Ferrous Ascorbate (N = 2). *One study reported the use of ferrous fumarate and one with parenteral iron polymaltose complex, so the meta-analysis of these individual studies could not be done, therefore the total included studies in this subgroup analysis (N = 26).

No significant heterogeneity was found between parenteral and oral routes (*p* = 0.73), suggesting that the route of administration is not a primary driver of variability. The detailed forest plot is shown in Fig 4. In this subgroup analysis, one study reported the use of ferrous fumarate and one with parenteral iron polymaltose complex, so the meta-analysis of these individual studies could not be done.

### Covariate analysis

Baseline Hb levels were inversely associated with Hb improvement (coefficient: -0.28, 95% CI: -0.92 to 0.36, *p* = 0.36). The combined visual depiction of improvement in Hb with other covariates is given in S1 Fig.

### Quality assessment and publications bias

Using JBI critical appraisal tools, nine studies achieved 90–100% quality criteria adherence, indicating a low risk of bias. Two studies [71, 84] had a high risk due to issues in allocation concealment and blinding. Publication bias was minimal, as indicated by symmetrical funnel plots and Egger’s test (*p* < 0.10) (S2 Fig).

### GRADE assessment

The overall quality of evidence for the pooled effect size estimates was rated moderate (3+). The use of ferrous sulfate was rated as high-quality evidence (4+). The moderate ratings reflect potential risks of bias primarily due to limitations in study design, such as a lack of blinding or allocation concealment. However, the included studies largely counteracted these risks by employing sufficient sample sizes and appropriate sampling methods. Details about the judgement of the quality of evidence factor for each outcome are provided in the S6 Table.

## Discussion

This systematic review and meta-analysis assessed the optimal dose and duration of iron supplementation for improving Hb levels in children and adolescents with uncomplicated IDA. A total of 8,829 participants from 28 studies were included, with a pooled effect size of 2.01 gm/dL (95% CI: 1.48–2.54, *p* < 0.001), demonstrating significant Hb improvement across intervention groups. Treatment duration influenced the effect size, with the highest improvement observed for treatments lasting less than three months (2.39 gm/dL, 95% CI: 0.72–4.07, *p* < 0.001). Treatments exceeding six months also showed notable efficacy, with an effect size of 1.93 gm/dL (95% CI: 0.09–3.77, *p* = 0.039). In contrast, interventions lasting three to six months exhibited a smaller improvement (1.58 gm/dL, 95% CI: 0.93–2.23, *p* < 0.001). These findings suggest that the benefits of iron supplementation may plateau during intermediate durations but increase marginally when treatment is extended. While no specific dose category achieved statistical significance, low-dose regimens (<5 mg/kg/day) exhibited favourable trend, particularly among individuals with lower baseline Hb levels. This indicates that combining low-dose supplementation with tailored shorter or extended durations could optimize Hb improvement.

Our findings align with previous studies that have examined similar parameters. For instance, Khosfetrat et al. (2012) demonstrated rapid increases in Hb and ferritin levels within 1.5 months of treatment, which stabilized thereafter, suggesting that longer treatment durations may yield diminishing returns [89]. Similarly, Akin et al. (2014) reported that parenteral iron sucrose improved ferritin levels in children with refractory IDA, although further increases in Hb levels plateaued over time. This observation highlights the potential risks of prolonged treatment, such as hyperferritinemia, and underscores the importance of monitoring iron supplementation to avoid adverse outcomes [90]. The results also corroborate the recommendations of India’s I-NIPI, which advocates a three-month duration of supplementation, extendable based on the severity of the deficiency [20, 27, 28]. The gradual slowing of Hb improvement over time may be attributable to various factors, including physiological adaptation, reduced patient compliance, or decreased iron absorption.

Our review confirmed that low-dose iron regimens (<5 mg/kg/day) are associated with improvement in Hb. For example, a trial reported that low-dose ferrous sulphate (3 mg/kg/day) was more effective in raising Hb concentration than iron polysaccharide complex [83]. In another study, micronutrient powders with standard (12.5 mg) or low-dose (5 mg) iron did not significantly differ in preventing anaemia but had fewer adverse effects, such as fever and diarrhoea [91]. Similarly, adding low doses of iron (5 or 10 mg/day) to school meals was shown to be equally effective in improving Hb and reducing anaemia in preschool children, reinforcing the utility of low-dose strategies in public health interventions [92].

Geographical differences in supplementation practices were evident and likely driven by variations in population characteristics, healthcare systems, and nutritional profiles. In LMICs, higher doses of iron (5–10 mg/kg/day) and longer treatment durations (over six months) were more common, addressing the severe deficiencies prevalent in these regions due to malnutrition and infectious diseases [93]. In contrast, HICs predominantly used lower doses (<5 mg/kg/day) and shorter treatment durations (less than three months) [83], reflecting milder deficiencies and more robust healthcare systems that enable timely interventions. Socioeconomic conditions, cultural dietary habits, and healthcare resources further contribute to these regional disparities. Populations in LMICs often consume diets severely deficient in iron, necessitating prolonged supplementation to achieve therapeutic goals, while superior healthcare infrastructure and resources in HICs make parenteral iron infusion a more feasible option. These findings emphasize the importance of tailoring iron supplementation strategies to regional needs, accounting for socioeconomic and cultural contexts, and evolving healthcare priorities.

Inorganic forms of iron, particularly ferrous sulfate, were the most commonly studied formulations and demonstrated significant efficacy, with oral ferrous sulfate reporting an effect size of 2.03 gm/dL (1.24–2.82, *p* < 0.001). Despite being less bioavailable and associated with greater gastrointestinal side effects compared to organic forms such as iron ascorbate or lysinate, inorganic formulations remain a pragmatic choice due to its affordability, availability, and inclusion in public health programs. This preference is supported by prior studies, such as those by Mahmood et al. (2017) and Power et al. (2017), which found ferrous sulfate more effective than iron polymaltose and polysaccharide complexes in raising Hb levels [83, 94].

Hb improvement did not differ significantly between ferrous sulphate and ferrous fumarate with zinc and vitamin C, further supporting the broad utility of ferrous sulfate [95]. The preference for ferrous sulfate in this review reflects its extensive documentation in clinical guidelines and its practical use in public health programs [96, 97]. Although the search strategy for this review included all forms of iron, the predominance of studies utilizing ferrous sulfate warranted a separate meta-analysis for this formulation. In contrast, the number of studies reporting the use of organic iron preparations was relatively low, prompting us to combine these formulations under a general ‘other’ category and conduct the meta-analysis accordingly. Thus, the limited number of studies employing organic iron formulations restricted our ability to conduct a robust comparative analysis. Future research should prioritize comparative trials to evaluate the efficacy, safety, and cost-effectiveness of organic iron formulations.

Baseline Hb levels were inversely associated with Hb improvement, with the effect size decreasing by 0.28 units for every unit increase in baseline Hb (β = -0.28, *p* = 0.36). This "ceiling effect" suggests that individuals with severe anaemia experience greater gains in Hb from supplementation, while those with near-normal levels exhibit limited improvements. This phenomenon, previously documented in similar studies, highlights the importance of stratifying treatment based on anaemia severity to achieve optimal outcomes [98–101].

This review has several strengths. It is the first to comprehensively evaluate the effects of iron supplementation dose and duration in children and adolescents, potentially informing future policy decisions regarding IDA management. The inclusion of mostly RCTs increased the validity of the findings, while substantial sample sizes enhanced their generalizability. Adherence to PRISMA guidelines and the application of GRADE assessment ensured methodological rigor and high-quality evidence. However, this review also has limitations. The heterogeneity of the included studies, which spanned diverse populations, limited the ability to conduct detailed age-specific analyses or stratify results by anaemia severity. Additionally, the interaction between dose escalation and treatment duration was not explicitly examined, leaving a critical gap in understanding the optimal balance between these factors. The limited representation of organic iron formulations also restricted the scope of the analysis, and inconsistent reporting of outcomes such as ferritin levels and adverse effects reduced the comprehensiveness of the findings.

Addressing anaemia in children and adolescents is critical not only for individual health outcomes but also for broader societal benefits, including improved educational attainment, workforce productivity, and national economic growth. Effective anaemia management aligns with global health objectives, such as the Sustainable Development Goals (SDGs) related to health (SDG 3) and education (SDG 4) [102, 103]. Governments should prioritize IDA reduction by integrating routine screening into national health policies and investing in public health programs that promote dietary diversity, nutritional education, and widespread iron supplementation. In resource-limited settings, cost-effective tools such as point-of-care Hb meters and Hb colour scales should be adopted to facilitate early diagnosis and monitoring of anaemia [104–106]. The findings of this review provide valuable guidance for developing region-specific guidelines and strengthening global efforts to reduce the burden of IDA, fostering sustainable development and enhancing the quality of life for affected populations.

## Conclusion

Low-dose iron supplementation (<5 mg/kg/day) effectively improves Hb levels in children and adolescents, particularly those with lower baseline Hb. Treatment durations of less than three months achieve the highest Hb increase (2.39 gm/dL), while extended regimens beyond six months yield 1.93 gm/dL improvement. Developing region-specific guidelines that prioritize low-dose strategies with appropriate durations is essential for ensuring equitable management of IDA.

## Supporting information

S1 Table Preferred Reporting Items for Systematic Reviews and Meta-Analyses (PRISMA) checklist. (DOCX)

S2 Table Search strategy for estimating optimal dose and duration of iron supplementation for treatment and maintaining recovery from IDA among children and adolescents. (DOCX)

S3 Table Quality assessment of individual studies using JBI checklist. (DOCX)

S4 Table List of all the studies identified from literature search along with the reasons for exclusion. (XLSX)

S5 Table Data extraction sheet for all the included studies. (XLSX)

S6 Table GRADE assessment for certainty of evidence. (DOCX)

S1 Fig Combined visual analysis of predictors and effect size using scatter plots with regression. (DOCX)

S2 Fig Funnel plot for estimation of publication bias. (DOCX)

## Abbreviations

CBC: Complete blood count
GRADE: Grading of Recommendations Assessment, Development and Evaluation
Hb: Haemoglobin
IDA: Iron Deficiency Anaemia
I-NIPI: Intensified National Iron Plus Initiative
JBI: Joanna Briggs Institute
LMICs: Low and Lower-Middle-Income Countries
NCHS: National Centre for Health Statistics
PRISMA: Preferred Reporting Items for Systematic Reviews and Meta-Analyses
RCTs: : Randomised controlled trials
SDGs: Sustainable development goals
WHO: World Health Organization
