# Supplementary material for: Optimal dose and duration of iron supplementation for treating iron deficiency anaemia in children and adolescents: A systematic review and meta-analysis
Source: PLoS One. 2025 Feb 14;20(2):e0319068. doi: 10.1371/journal.pone.0319068 (PMC11828412; doi:10.1371/journal.pone.0319068)
Supplement: S2 Table — (DOCX) [file pone.0319068.s002.docx]

**S2 Table. Search strategy for estimating optimal dose and duration of iron supplementation for treatment and maintaining recovery from IDA among children and adolescents**

| **Sr. No** | **Concept** | **Search strategy** |
| --- | --- | --- |
| **1** | **Child or Adolescent** | "child"[MeSH Terms] OR "adult children"[MeSH Terms] OR "child*"[Title/Abstract] OR "adolescent"[MeSH Terms] OR "adolescent"[MeSH Terms] OR "adolescen*"[Title/Abstract] OR "pediatric*"[Title/Abstract] OR "paediatric*"[Title/Abstract] OR "population"[MeSH Terms] OR "child health"[MeSH Terms] OR "child health service*"[MeSH Terms] OR "child, preschool"[MeSH Terms] OR "child, hospitalized"[MeSH Terms] OR "under five children"[Title/Abstract] OR "under 5 children"[Title/Abstract] OR "children 6 59 months old"[Title/Abstract] OR (("child"[MeSH Terms] OR "child"[All Fields] OR "children"[All Fields] OR "child s"[All Fields] OR "children s"[All Fields] OR "childrens"[All Fields] OR "childs"[All Fields]) AND "20 years"[Title/Abstract]) |
| **2** | **Anemia** | "anemia"[MeSH Terms] OR "anemia, iron deficiency"[MeSH Terms] OR "hematologic disease*"[MeSH Terms] OR "haemoglobinopathies"[Title/Abstract] OR "hematological profile"[Title/Abstract] OR "hematological abnormalities"[Title/Abstract] OR "hematological parameters"[Title/Abstract] OR "iron status"[Title/Abstract] OR (("iron deficiencies"[MeSH Terms] OR ("iron"[All Fields] AND "deficiencies"[All Fields]) OR "iron deficiencies"[All Fields] OR ("iron"[All Fields] AND "deficiency"[All Fields]) OR "iron deficiency"[All Fields]) AND "anemia*"[MeSH Terms]) OR "hematologic test*"[MeSH Terms] OR "nutritional status"[MeSH Terms] OR "hemoglobins"[MeSH Terms] OR "nutritional status"[MeSH Terms] OR "nutrition assessment"[MeSH Terms] OR "iron deficiencies"[MeSH Terms] |
| **3** | **Iron folic acid for treatment of anemia** | "iron dextran complex"[MeSH Terms] OR "Iron"[MeSH Terms] OR "iron, dietary"[MeSH Terms] OR "parenteral iron"[Title/Abstract] OR "oral iron"[Title/Abstract] OR "folic acid"[MeSH Terms] OR "IFA"[Title/Abstract] OR "iron folic acid"[Title/Abstract] OR "elemental iron"[Title/Abstract] OR "ifa supplements"[Title/Abstract] OR "iron regimens"[Title/Abstract] OR "micronutrients"[MeSH Terms] OR "ferrous compounds"[MeSH Terms] OR "ferrous sulfate"[Title/Abstract] OR "ferrous fumarate"[Title/Abstract] OR "ifa syrup"[Title/Abstract] OR "Time-to-Treatment"[Title/Abstract] OR "treatment"[Title/Abstract] OR "Time-to-Treatment"[MeSH Terms] OR "patient compliance"[MeSH Terms] OR "compliance"[MeSH Terms] OR "treatment adherence and compliance"[MeSH Terms] OR "guideline adherence"[MeSH Terms] OR "patient compliance"[MeSH Terms] OR "medication adherence"[MeSH Terms] |
| **4** | **Combined** | #1 AND #2 AND #3 |
