# Supplementary material for: Optimal dose and duration of iron supplementation for treating iron deficiency anaemia in children and adolescents: A systematic review and meta-analysis
Source: PLoS One. 2025 Feb 14;20(2):e0319068. doi: 10.1371/journal.pone.0319068 (PMC11828412; doi:10.1371/journal.pone.0319068)
Supplement: S3 Table — (DOCX) [file pone.0319068.s003.docx]

| **JBI’s critical appraisal tool for quality assessment for Randomized Controlled Trials (RCTs)** | | | | | | | | | | | | | | | |
| --- | --- | --- | --- | --- | --- | --- | --- | --- | --- | --- | --- | --- | --- | --- | --- |
| **SN** | **Author name, Year** | **Was true randomization used for assignment of participants to treat groups?** | **Was allocation to treatment groups concealed?** | **Were treatment groups similar at the baseline?** | **Were participants blind to treatment assignment?** | **Were those delivering treatment blind to treatment assignment?** | **Were outcomes assessors blind to treatment assignment?** | **Were treatment groups treated identically other than the intervention of interest?** | **Was follow up complete and if not, were differences between groups in terms of their follow up adequately described and analysed?** | **Were participants analysed in the groups to which they were randomized?** | **Were outcomes measured in the same way for treatment groups?** | **Were outcomes measured in a reliable way?** | **Was appropriate statistical analysis used?** | **Was the trial design appropriate, and any deviations from the standard RCT design (individual randomization, parallel groups) accounted for in the conduct and analysis of the trial?** | **%** |
| 1 | Pasricha et al, 2017 | Yes | Yes | No | Yes | Yes | No | Yes | Yes | Yes | Yes | Yes | Yes | Yes | 85% |
| 2 | Falahati et al, 2020 | Yes | NA | Yes | No | No | No | Yes | Yes | Yes | Yes | Yes | Yes | Yes | 77% |
| 3 | Fujita et al, 2017 | Unclear | NA | Yes | NA | NA | Yes | Yes | Yes | Yes | Yes | Yes | Yes | Yes | 77% |
| 4 | Bah & Stelle et al, 2021 | Yes | Yes | Yes | Yes | Yes | Yes | Yes | Yes | Yes | Yes | Yes | Yes | Yes | 100% |
| 5 | Moradveisi et al, 2019 | Unclear | NA | Yes | NA | NA | NA | Yes | NA | Yes | Yes | Yes | Yes | Yes | 70% |
| 6 | Svensson et al, 2015 | Yes | Yes | Yes | Yes | Yes | Yes | Yes | Yes | Yes | Yes | Yes | Yes | Yes | 100% |
| 7 | Varea et al, 2017 | Yes | Yes | Yes | Yes | No | Yes | Yes | Yes | Yes | Yes | Yes | Yes | Yes | 93% |
| 8 | Chirdkiatgumchai et al, 2016 | Unclear | Yes | Yes | Yes | Yes | NA | Yes | Yes | Yes | Yes | Yes | Yes | Yes | 93% |
| 9 | Amrousy et al, 2020 | Yes | Yes | Yes | NA | NA | NA | Yes | Yes | Yes | Yes | Yes | Yes | Yes | 77% |
| 10 | Hamed et al, 2019 | Unclear | NA | Yes | NA | NA | NA | Yes | Yes | Yes | Yes | Yes | Yes | Yes | 70% |
| 11 | Gupta et al, 2013 | Yes | Yes | Yes | NA | NA | NA | Yes | Yes | Yes | Yes | Yes | Yes | Yes | 77% |
| 12 | Gupta et al, 2017 | Yes | No | Yes | Yes | No | No | Yes | Yes | Yes | Yes | Yes | Yes | Yes | 77% |
| 13 | Bakht et al, 2022 | Yes | NA | Yes | NA | NA | NA | Yes | Yes | Yes | Yes | Yes | Yes | Yes | 70% |
| 14 | Mohammed et al, 2017 | Yes | Yes | Yes | Yes | Yes | Yes | Yes | Yes | Yes | Yes | Yes | Yes | Yes | 100% |
| 15 | Yun et al, 2020 | No | No | No | No | NA | NA | Yes | Yes | Yes | Yes | Yes | Yes | Unclear | 70% |
| 16 | Kamal et al, 2021 | Unclear | NA | Yes | NA | NA | NA | Yes | Yes | Yes | Yes | Yes | Yes | Yes | 70% |
| 17 | Fisyunn et al, 2019 | Yes | NA | Yes | Unclear | NA | NA | Yes | Yes | Yes | Yes | Yes | Yes | Yes | 70% |
| 18 | Powers & Mark et al, 2015 | No | No | No | NA | NA | NA | Yes | Yes | Yes | Yes | Yes | Yes | Yes | 77% |
| 19 | Matos et al, 2016 | Yes | Unclear | Yes | Unclear | Yes | Yes | Yes | Yes | Yes | Yes | Yes | Yes | Yes | 85% |
| 20 | Kaushik et al, 2020 | Yes | NA | Yes | NA | NA | NA | Yes | Yes | Yes | Yes | Yes | Yes | Yes | 70% |
| 21 | Powers & George et al, 2015 | Yes | Yes | Yes | Yes | Yes | No | Yes | Yes | Yes | Yes | Yes | Yes | Yes | 93% |
| 22 | Korczowski et al, 2015 | No | No | Unclear | No | No | No | Yes | Yes | Yes | Yes | Yes | Yes | Yes | 70% |
| 23 | Patil et al, 2016 | Yes | Yes | Yes | No | No | No | Yes | Yes | Yes | Yes | Yes | Yes | Yes | 77% |
| 24 | Wegier et al, 2016 | No | No | Yes | NA | NA | NA | Yes | Yes | Yes | Yes | Yes | Yes | Yes | 85% |
| 25 | Name et al, 2016 | Yes | Unclear | Yes | Yes | NA | Yes | Yes | Yes | Yes | Yes | Yes | Yes | Yes | 93% |

| **JBI’s critical appraisal tool for quality assessment for Cohort studies** | | | | | | | | | | | | | |
| --- | --- | --- | --- | --- | --- | --- | --- | --- | --- | --- | --- | --- | --- |
| **SN** | **Author name, Year** | **Were the two groups similar and recruited from the same population?** | **Were the exposures measured similarly to assign people to both exposed and unexposed groups?** | **Was the exposure measured in valid and reliable way?** | **Were confounding factors identified?** | **Were strategies to deal with confounding factors stated?** | **Were the groups/participants free of the outcome at the start of the study (or at the moment of exposure)?** | **Were the outcomes measured in a valid and reliable way?** | **Was the follow up time reported and sufficient to be long enough for outcomes to occur?** | **Was follow up complete, and if not, were the reasons to loss to follow up described and explored?** | **Were strategies to address incomplete follow up utilized?** | **Was appropriate statistical analysis used?** | **%** |
| 1 | Sourabh et al, 2018 | Yes | NA | NA | NA | NA | No | Yes | Yes | Yes | No | Yes | 93% |
| 2 | Azevedo et al, 2014 | Yes | NA | Yes | NA | NA | No | Yes | Yes | Yes | Yes | Yes | 70% |
| 3 | Gosdin et al, 2015 | Yes | Yes | Yes | Yes | Yes | Yes | Yes | Yes | Yes | Yes | Yes | 100% |
