# Supplementary material for: Optimal dose and duration of iron supplementation for treating iron deficiency anaemia in children and adolescents: A systematic review and meta-analysis
Source: PLoS One. 2025 Feb 14;20(2):e0319068. doi: 10.1371/journal.pone.0319068 (PMC11828412; doi:10.1371/journal.pone.0319068)
Supplement: S6 Table — (DOCX) [file pone.0319068.s006.docx]

| **Certainty assessment** | | | | | | | **Summary of findings** | |
| --- | --- | --- | --- | --- | --- | --- | --- | --- |
| **No of studies** | **Study design** | **Risk of bias** | **Inconsistency** | **Indirectness** | **Imprecision** | **Other considerations** | **Effect** | **Certainty** |
|  |  |  |  |  |  |  | **Absolute**  **(95% CI)** |  |
| **Improvement in Hb (assessed with: SMD)** | | | | | | | | |
| 28 | Randomised trials | Not serious | Serious | Not serious | Not serious | None | SMD 2 SD higher  (1.49 higher to 2.5 higher) | ⨁⨁⨁◯ Moderate |
| **Hb supplementation <3 months (assessed with: SMD)** | | | | | | | | |
| 13 | Randomised trials | Not serious | Serious^a^ | Not serious | Not serious | None | SMD 2.39 SD higher  (0.72 higher to 4.07 higher) | ⨁⨁⨁◯ Moderate ^a^ |
| **Hb supplementation 3-6 months (assessed with: SMD)** | | | | | | | | |
| 13 | Randomised trials | Not serious | Serious^b^ | Not serious | Not serious | None | SMD 1.58 SD higher  (0.93 higher to 2.23 higher) | ⨁⨁⨁◯ Moderate ^b^ |
| **Hb supplementation >6 months (assessed with: SMD)** | | | | | | | | |
| 2 | Randomised trials | Not serious | Serious^b^ | Not serious | Not serious | None | SMD 1.93 SD higher  (0.09 higher to 3.76 higher) | ⨁⨁⨁◯ Moderate ^b^ |
| **Ferrous Sulfate (assessed with: SMD)** | | | | | | | | |
| 14 | Randomised trials | Not serious | Not serious | Not serious | Not serious | None | SMD 2.02 SD higher  (1.23 higher to 2.81 higher) | ⨁⨁⨁⨁ High |

**S6 Table. GRADE assessment for certainty of evidence**
