# Supplementary material for: Optimal dose and duration of iron supplementation for treating iron deficiency anaemia in children and adolescents: A systematic review and meta-analysis
Source: PLoS One. 2025 Feb 14;20(2):e0319068. doi: 10.1371/journal.pone.0319068 (PMC11828412; doi:10.1371/journal.pone.0319068)
Supplement: S1 Fig — (DOCX) [file pone.0319068.s007.docx]

**S1 Fig. Combined visual analysis of predictors and effect size using scatter plots with regression**

**
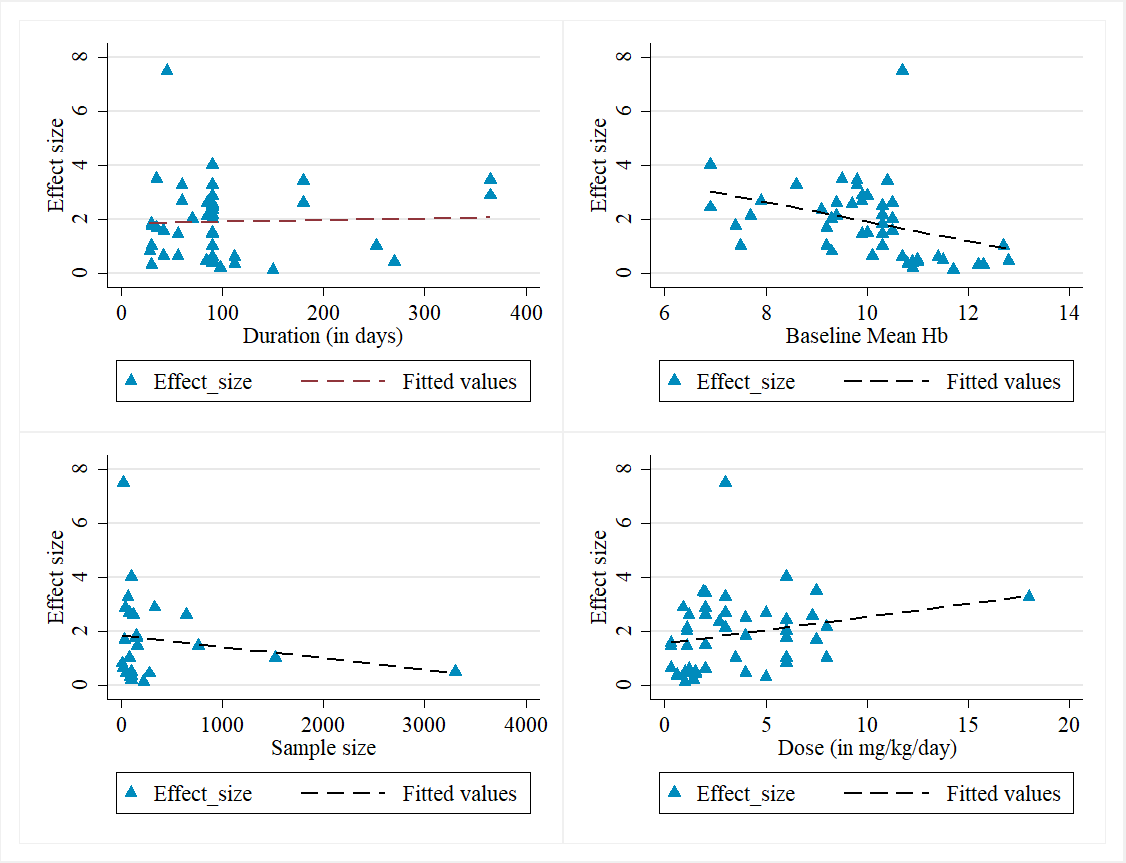
**
