# Supplementary figures and images for: Optimal dose and duration of iron supplementation for treating iron deficiency anaemia in children and adolescents: A systematic review and meta-analysis
Source: PLoS One. 2025 Feb 14;20(2):e0319068. doi: 10.1371/journal.pone.0319068 (PMC11828412; doi:10.1371/journal.pone.0319068)

**S2 Fig. Funnel plot for estimation of publication bias**


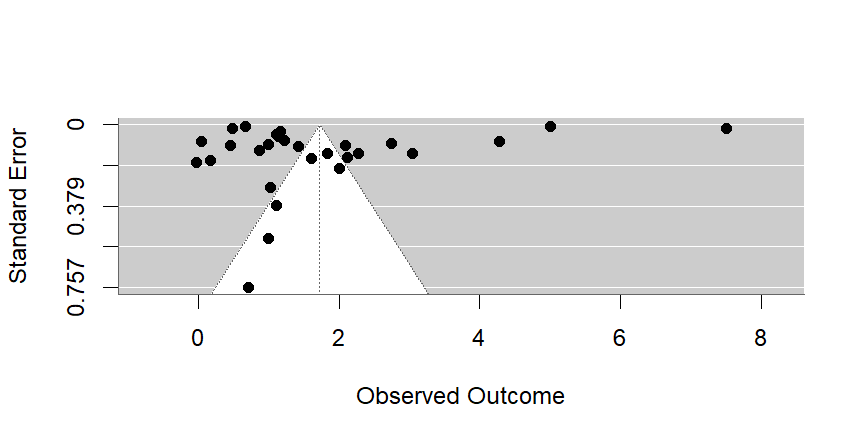

Supplement: S2 Fig — (DOCX) [file pone.0319068.s008.docx]
